# The influence of transportation, social norms, cultural identity, and affective disposition in transnational media enjoyment

**DOI:** 10.3389/fpsyg.2024.1377898

**Published:** 2024-11-21

**Authors:** Jing Wang, Qiqi Ye, Zhiqiang Shuai, Peifeng Wang, Yujie Wang, Changqing Lin

**Affiliations:** ^1^School of Culture and Communication, Putian University, Putian, China; ^2^School of Literature and Communication, Wenzhou University of Technology, Wenzhou, China; ^3^Faculty of Language and Communication, Sultan Idris Education University, Tanjong Malim, Malaysia; ^4^School of Arts and Designs, Putian University, Putian, China

**Keywords:** transportation, international TV dramas, enjoyment, social norms, cultural identity, affective disposition

## Abstract

**Background:**

The globalization of the media market is forcing decision-makers to understand the psychological processes behind local audiences’ enjoyment of foreign TV dramas. Transportation is a well-established psychological theory and framework utilized to elucidate and anticipate audience engagement and enjoyment in the cognitive process of experiencing a narrative text. Although there is a substantial body of literature on transportation and media enjoyment, there is a noticeable absence of studies on the relationship between audiences being “transported” into the narrative world of TV dramas and, particularly, the pleasure audiences derive from interacting with media content within a cross-cultural acceptance context.

**Method:**

The research employs a quantitative design, with responses collected from 353 students enrolled at a Malaysian public university. It aims to validate the influence of social norms, cultural identity, and affective disposition on narrative immersion while watching foreign TV dramas, as well as the subsequent enjoyment of media among local audiences.

**Results:**

The results indicate that social norms, cultural identity, and affective disposition significantly influence transportation and enjoyment. Furthermore, the influence of cultural identity on social norms has a positive moderating effect on transportation.

**Discussion:**

Storytelling that complies with social norms while offering new perspectives can maximally engage audiences, potentially altering their narrative cognition and deepening their immersion in fictional narratives. Cultural identity can shape audience perceptions and reactions to cross-cultural media consumption, ultimately influencing the degree to which audiences are drawn into the narrative. Furthermore, the audience’s emotional connection to characters in the narrative or to situations in the drama significantly influences the overall cognitive and immersion levels.

## 1 Introduction

Currently, watching foreign TV dramas is a popular way to understand global cultures. The popularity of foreign TV series reflects the cultural logic of media globalization (Nazri and Ahmad, 2022). This phenomenon is shaped by the competition and interaction between the audiences’ global media industry needs and culture in the context of the globalization of the media industry (Waisbord, 2004; Baek and Kim, 2016; Lu et al., 2019; Chan et al., 2021). Similarly, watching foreign TV dramas is considered to symbolize a fashionable lifestyle in Malaysia (Rahim, 2018). Foreign TV dramas, accompanied by the image of modern life and an exotic charm, have fulfilled the growing tastes of Malaysian audiences (Kang et al., 2022). These audiences crave prosperity, comfort, fashion, and hedonism (e.g., Lu et al., 2019; Jiang and Leung, 2012; Owen and Riggs, 2012; Jie Chan et al., 2022). Previous research on Malaysian audiences’ reception of foreign media products employed media content and audience characteristics to predict local audience preferences (e.g., Nabi and Krcmar, 2004; Budarick and Han, 2018; Loke and Bahiyah, 2020) or has utilized uses and gratifications theory to explain why and how audiences are attracted to foreign TV dramas (Khai and Wahab, 2017). Furthermore, studies have shown that a wide variety of novel characters, situations, and background scenes (Vorderer and Hartmann, 2009; Tallón-Ballesteros, 2023) may rouse the interest or curiosity of the viewers, thereby contributing to the tendency of viewers to seek psychological pleasure from media entertainment (Baek and Kim, 2016). Additionally, some studies have also discussed the considerable influence of genre on audience experience and enjoyment (Bordun, 2017; Thompson et al., 2021). Cultural proximity (Yim and Kang, 2018) and genre proximity (Eriko, 2012) are often employed to predict the acceptance of transnational TV dramas. However, few studies have examined the mechanisms by which viewers derive enjoyment by engaging with media content while watching foreign TV dramas or the viewing process (Lu et al., 2019).

Enjoyment is the core of media entertainment, referring to the result of the experience of watching (Xu and Yan, 2011; Lu et al., 2019; Xu and Yan, 2011) or consuming media content (Vorderer et al., 2004). It is commonly described as a general positive disposition and preference towards media content (Lu et al., 2019; Nabi and Krcmar, 2004). The current literature presents psychological mechanisms that elucidate the enjoyment enhancement effect experienced by local audiences when viewing international TV programs (McFadyen et al., 2000; Vorderer and Hartmann, 2009). Certain models emphasize social determinants that forecast enjoyment, including social norms (SN) and viewing contexts (e.g., Denham, 2004; McGloin et al., 2016). Local audiences’ appreciation of foreign TV drama is impacted. Additional research examines the perception of identity and novelty as both opposing and coexisting elements, analyzing the dual effects of “inhibiting enjoyment” and “enhancing enjoyment” on the media enjoyment of local audiences viewing foreign TV dramas (Baek and Kim, 2016; Vorderer and Hartmann, 2009). Nonetheless, in examining the correlation between international TV dramas and viewers, a complete theoretical model that encompasses the psychological processes of perception and enjoyment among local audiences is lacking (Baek and Kim, 2016).

TV drama, as a genre of drama, is typically conveyed to the audience through storytelling, with the primary objective of immersing the audience in the storyline. Scholars of narrative immersion assert that “narrative” presentation engenders audience engagement with the story, facilitating enjoyment through resonance with characters or ideas (e.g., Green et al., 2004; Busselle and Bilandzic, 2008; Tamborini et al., 2021a, 2021b). Transportation is a well-established psychological theory and framework utilized to elucidate and anticipate audience engagement and enjoyment in the cognitive process of experiencing a narrative text (Eyal and Rubin, 2003; Escalas, 2007; Slater et al., 2006; Lu et al., 2019; Sadeghzadeh Fesaghandis, 2021; Thompson et al., 2021). This study posits that transport theory serves as an optimal framework for examining the psychological processes of local audiences engaging with foreign TV drama. Although there is a substantial body of literature on transportation and media enjoyment (Owen and Riggs, 2012; Vorderer et al., 2004; Acic, 2023), there is a noticeable absence of studies on the relationship between audiences being ‘transported’ into the narrative world of TV dramas and media enjoyment within a cross-cultural acceptance context (Owen and Riggs, 2012; Krause, 2020). Furthermore, theoretically driven research on transnational media enjoyment is limited in Malaysia. Firstly, most studies in the literature review are confined to Korean TV dramas (Soo and Jamil, 2022; Loke and Bahiyah, 2020; Keane, 2019; Kim et al., 2019). Secondly, there are few studies that are set against the background of Mainland Chinese TV dramas (Huang et al., 2021; Kang et al., 2020).

In this regard, we need to develop a more comprehensive model to analyze the factors that influence audiences’ transportation into the narrative world of TV dramas in the context of transnational cultural acceptance. First, we consider social contexts, which can influence an individual’s media choices and evaluations (Lu et al., 2019; Kryston and Eden, 2022). However, media scholars have yet to adopt (Owen and Riggs, 2012) or create a coherent theory to explain how group membership influences an individual’s media choices and enjoyment (Kryston and Eden, 2022; Sherry, 2004). Considering the role of SN and their impact on attitudes or perceptions within specific social groups (Chung and Rimal, 2016; Hogg et al., 2017; Liu, 2022) may help us understand how others’ preferences regarding transnational media products affect an individual’s media enjoyment (Kryston and Eden, 2022). Therefore, we introduce a framework based on perceived norms to understand media evaluations and choices.

Secondly, Oatley and Johnson-Laird (1996) suggested that emotional experiences are one of the reasons why people choose to immerse themselves in narrative fiction. Nevertheless, this study aims to elucidate the relationship between audiences’ emotional tendencies (such as emotional empathy toward the character) and narrative immersion and media enjoyment (Black et al., 2019; Walkington et al., 2020). Thirdly, we target different cultures, as culturally targeted narratives are often used as an informational strategy among minority groups, including African Americans, Latinos, and Asians (Tan, 2021; Hinyard and Kreuter, 2007). Few studies have focused on the relationship between cultural factors [such as familiarity with the cultural background of the narrative and cultural identity (CI) and their influence on transportation (e.g., Cohen and Shrum, 2001; Bilandzic and Busselle, 2011)].

Therefore, research integrating transportation theory, a framework based on perceived norms, and theories of CI in cross-cultural communication still needs to be explored. Accordingly, this study employs the theoretical framework of transportation to expand SN, CI, and affective disposition (AD) into a conceptual model for testing the formulated hypotheses.

This study presents and empirically examines a model that identifies the factors influencing narrative immersion and media enjoyment among audiences when watching foreign TV dramas. These factors impact the audiences’ personal experience of the narrative and overall viewing experience, subsequently affecting their enjoyment. The significance of this study lies in its comprehensive framework, which examines the relationship between narrative and media enjoyment based on perceptual norms, cultural/socio-cultural factors, and personal emotional tendencies. This facilitates the explanation and understanding of audiences’ comprehension, immersion, and enjoyment of media narrative content across cultures. The purpose of this empirical application is to provide academic integrity, guidance, and suggestions for predicting individual narrative experiences and enjoyment during the process of watching transnational TV dramas. It aims to attract more attention from researchers in the fields of psychology and media. Therefore, the main objective of this study is to use the transportation model as a theoretical framework to test Malaysian audiences’ media enjoyment when watching Chinese TV dramas and to investigate the relationship between the components.

## 2 Literature review and hypotheses development

### 2.1 Theoretical foundation

To explore audiences’ enjoyment of the story world, scholars have focused on the narrative experience itself (Green et al., 2004; Green, 2008; Escalas, 2007; Slater et al., 2006). Subsequently, scholars shifted their attention to the narrative experience to assess the degree of enjoyment audiences derive from the story world (Kim, 2020), with other researchers also confirming this (Jang et al., 2016; Jang et al., 2016; Green et al., 2004; Escalas, 2007). Green and Brock (2000) were the first to develop a structural model of the transportation scale to explain the cognitive and emotional immersion of audiences or readers when engaging with text-based narratives (Busselle and Bilandzic, 2009).

Transportation refers to the metaphorical emotional construct presented in the narrative. The theoretical basis of transportation in mental imagery has been confirmed multiple times (Owen and Riggs, 2012; Green and Brock, 2002). The pursuit of enjoyment typically motivates readers and audiences to immerse themselves in the narrative world (Green, 2004; Owen and Riggs, 2012; Budelmann et al., 2017). Transportation is a three-dimensional concept that requires comprehensive engagement of audiences’ attention, emotions, and imagination (Green et al., 2004). Green and Brock (2000) explained that audiences must undergo three basic processes to immerse themselves fully. Initially, audiences must possess a wealth of cognitive resources to effectively digest information from the fictional domain (Kuijpers et al., 2014; McPhail, 2022). Then, imaginative traits lead to vivid mental images generated by the narrative, resulting in an immersive experience for the listener (Kuhne, 2017). Finally, by empathizing with the characters, audiences can understand what they are going through and experience their emotions and perspectives.

Furthermore, previous studies have shown that cognitive engagement, emotional engagement (e.g., Green and Brock, 2000; Appel and Richter, 2010; Mazzocco et al., 2010; Thompson et al., 2021), identification (Kryston and Eden, 2022), arousal transfer (Zillmann, 2003), and emotional response (Hoffner, 2009; Lee, 2015) can all influence how much people enjoy a film (Thelen et al., 2020). However, there has not been much systematic study on why viewers watch foreign TV dramas, particularly in identifying the cognitive and psychological factors involved (Baek and Kim, 2016; Jensen, 2017). Considering the ubiquity of audiovisual narratives in our culture, this may come as a surprise (Owen and Riggs, 2012; Malone and Cuddy, 2018).

Identification with a character is difficult to separate from the environment and situation in which the character is situated (Cohen, 2001). In both the Entertainment Overcoming Resistance Model (EORM; Moyer-Guse, 2008; Tan, 2021) and the Narrative Effects for Cultural Targeting Model (NECT; Tan, 2021), cultural variables are considered important factors in the psychological process of narrative construction. Additionally, the models above include a positive influence of sociocultural variables across cultural pairs. To expand the explanatory power of transportation, this study integrates a perception-based normative framework, EORM, and NECT.

### 2.2 Social norms and transportation

The concept of SN can be defined as the perception of an individual’s behavior (Owen and Riggs, 2012; Ajzen and Fishbein, 1977) or thoughts as they relate to the behavior or thoughts of most people in a group (Lapinski and Rimal, 2005; Kryston and Eden, 2022). Although definitions in the literature are inconsistent (Shulman et al., 2017), recent studies explicitly define SN as the consensus on behaviors (or behavioral approval) among multiple members of a reference group (Kryston and Eden, 2022). This means individuals may not know if their behavior is “correct” (Cialdini, 1993). However, they are more inclined to conform to the perceived expectations of others (Rhodes et al., 2020; Kryston and Eden, 2022) or to the perceived norms they ultimately measure (Chung and Rimal, 2016). Therefore, most research on SN relies on participants’ perceptions of SN or normative behavior since actual norms are difficult to quantify and measure (Shulman et al., 2017; Mehmood et al., 2024). Evidence suggests that under the influence of factors such as consensus, specific group affiliations, and social context, individuals tend to act according to what they believe others expect of them (Kryston and Eden, 2022) because SN guides individuals to act according to what they consider to be the group’s dominant behavior (Owen and Riggs, 2012; Rhodes et al., 2020).

The impact of SN on media selection and processing is well-established in the literature (Christopher, 2021), with studies confirming the theoretical underpinnings of media psychology (Katz et al., 1974; Trepte, 2006; Waddell and Sundar, 2020). Therefore, our study aligns with the research by Kryston and Eden (2022), which advocates for the expansion of the SN concept into perceived SN to promote the practical examination of audiences’ narrative immersion and media enjoyment.

The influence of SN on media selection and processing has been confirmed in the literature, with research primarily focusing on how consensus, group identification, and social context (e.g., being in a crowd) lead individuals to make consistent choices and experience enjoyment (Christopher, 2021). Previous studies have confirmed the influence of SN on media exposure and enjoyment (e.g., Katz et al., 1974; Trepte, 2006; Waddell and Sundar, 2020). Simply put, individuals are more likely to follow the norms of their close friends, family, or school/workplace (Borsari and Carey, 2003; Kryston and Eden, 2022; Hanich, 2022). For example, media selection may be influenced by perceptions of what content other group members are consuming (Katz et al., 1974; Park et al., 2020; Cummings et al., 2020).

Media scholars believe that perceived SN influences a person’s decision to watch movies or TV shows by providing heuristic cues, such as the idea that others are doing it (Chung and Rimal, 2016) or recommendations from close groups (e.g., friends, family, fraternities/sororities). Alternatively, people choose to watch when they perceive that others in the group approve of it (Johnson and Ranzini, 2018). It has been shown that familiarity plays a significant role in transportation (Green et al., 2008; Vaughn et al., 2010). According to Johnson and Rosenbaum (2018), friend recommendations and exhortations from close friends can also positively affect transportation since they can increase the viewer’s familiarity with the TV series and allow them to process the narrative more fluently (Rosenbaum and Johnson, 2016). Additionally, previous studies contend that social groups may incorporate perceived norms into the interpretive frameworks of stories (Pressgrove and Bowman, 2021), thereby converting stories into narratives reflective of their own identities. Consequently, when a social group disseminates or endorses a narrative text, it incorporates additional pre-story interpretative strategies, rendering the audience less receptive to adverse conditions prior to story acceptance and thereby diminishing the effect on narrative transportation (Van Laer et al., 2014).

However, past research has primarily focused on the impact of individual cognitive needs on transportation (Owen and Riggs, 2012; Giles and Maltby, 2002), with less attention given to how collective opinions affect media enjoyment (Kryston and Eden, 2022); the potential influence of the link between perceived SN and narrative transportation has also not been addressed (Van Laer et al., 2014). Therefore, this study posits that SN contributes to engaging the audience in the narratives of different media, thereby influencing their media enjoyment. Based on this, the hypothesis is as follows:

*Hypothesis 1:* Social norms are positively associated with transportation.

### 2.3 Cultural identity and transportation

The concept of CI can be defined broadly as “the identification and acceptance of a shared system of symbols and meanings” (Collier and Thomas, 1988) and the attachment to one’s cultural group (Mastro and Greenberg, 2000). In contrast to other physical goods, cultural goods are associated with SN values and the country in which they are rooted (Baek and Kim, 2016; Elasmar, 2014). Transnational TV dramas offer international audiences the opportunity to experience local culture, mainstream culture, and global consumer culture through cross-cultural exchange (Baek and Kim, 2016). By watching foreign TV dramas, the audience encounters the foreign values or norms embedded in the product, resulting in a mediated cross-cultural experience of CI (Katz and Liebes, 1990).

The idea behind this study is that CI is when people internalize a cultural worldview or framework and become familiar with, understand, and interpret the values, norms, and goals of other cultures while interacting and negotiating with people from other cultures (Liu, 2017). Furthermore, immigration, ethnicity, and cultural connection were not covered in the study. When the concept of “cultural identity” is introduced, it refers to the perception, acceptance, and understanding that occur throughout the cross-cultural interaction process between local audiences and the content of foreign media products in the age of media globalization.

Therefore, we utilize the above concept of CI, specifically focusing on the identification and understanding of Chinese culture among Malaysian audiences. This approach is particularly useful in analyzing the relationship between local Malaysian audiences’ recognition and understanding of Chinese culture, transportation, and media consumption in the context of transnational viewing.

A previous study has confirmed a direct relationship between the strength of a person’s identification and their entertainment choices (Trepte, 2006). An individual’s CI can significantly influence their perceptions and reactions to cross-cultural media consumption. In turn, this can affect their degree of narrative engagement (Cohen, 2001; Tamborini and Weber, 2020). Kim et al. (2016) asserted that cultural distance does not impede the transportation process in cross-cultural advertising narratives. Zhao and Liu (2018) argued that CI plays an important role in determining the extent of audience involvement with characters and stories in international TV dramas. Kryston and Eden (2022) found that consensus identification with other cultures positively affects media choice and narrative immersion. Huang et al. (2021) argued CI significantly influences individuals’ views and attitudes toward entertainment content (Raney and Ji, 2017), particularly regarding the ability to comprehend media content. Waddell and Sundar (2020) pointed out that CI significantly influences engagement with cross-cultural media, especially among individuals with a strong sense of belonging to a particular culture. A study by Tiede and Appel (2020) asserted that audiences are more likely to enjoy narratives and characters that reflect their cultural background since the social factors the audience perceives contribute to the level of enjoyment and resonance of the narrative. Larkey and Hecht (2010) confirmed that CI plays a significant role in narrative transmission. Tan (2021) indicated that within narrative contexts involving CI, individuals with bicultural backgrounds exhibit a higher propensity for narrative transportation.

Based on this, we propose the following hypothesis:

*Hypothesis 2:* Cultural identity is positively associated with transportation.

### 2.4 Affective disposition and transportation

Affective Disposition Theory (ADT) is a prominent theory within the field of narrative reception theory (Zillmann and Bryant, 1975; Zillmann and Cantor, 1976; Zillmann, 2003; Thompson et al., 2021; Grizzard et al., 2023). ADT seeks to explain the emotional responses of audiences to characters and their outcomes in fictional narratives, positing that pleasure is derived when characters receive the rewards they merit (Raney, 2003; Klimmt and Vorderer, 2003; Schibler, 2022). Its primary function is to elucidate the process of engaging with media narratives (Raney, 2004) and has garnered significant recognition within the academic community (Oliver et al., 2019). ADT posits that the audience continuously monitors the characters and their behavior, meaning the intensity of the audience’s emotional attachment to these characters may change over the course of the narrative, potentially impacting enjoyment (Raney, 2004). Zillmann and Cantor (1976) proposed that our emotional reactions to characters in a narrative, especially the main characters, significantly influence our overall appreciation of the narrative (Zillmann, 2003). Additionally, other research indicates that experiencing a narrative may alter responses to others’ AD (e.g., Paluck, 2009; Walkington et al., 2020; Grizzard et al., 2020). As an important theoretical pillar in entertainment theory, ADT simulates our emotional responses to characters and their fates within fictional narratives (Owen and Riggs, 2012; Grizzard et al., 2020). Therefore, this study incorporates ADT into the transportation model and investigates the role of AD as a variable influencing audience narrative immersion when watching international TV dramas.

Raney (2004) posited that audience’s knowledge and experience serve as the foundation for evaluating characters. Green et al. (2004) posited that audience immersion in a narrative necessitates a connection with the characters. When the audience cultivates familiarity with the actors in the play, it facilitates deeper engagement with the story (Moyer-Guse, 2008) and enhances enjoyment (Raney, 2004). Owen and Riggs (2012) suggested that the construct of identification resembles AD because the audience’s complex emotions toward the character create a concept called “involvement with the character” (Busselle and Bilandzic, 2008; Murphy et al., 2011). For example, Green et al. (2004) suggested that the enjoyment generated by transportation may be mediated by identification; Tal-Or and Cohen (2010) emphasized that people’s identification with a role enhances their enjoyment of transportation. By identifying with the characters, the audience can become immersed in their stories, whereas transportation (such as the sensory immersive experience provided by IMAX theaters) helps the audience identify with the film’s characters (Owen and Riggs, 2012; Perse, 1998). The aforementioned perspectives suggest that these constructs can affect each other or be used interchangeably (Owen and Riggs, 2012; Godlewski and Perse, 2010).

Moreover, individual differences in emotional processing can influence the level of immersion in a story and the resulting transportation effects (Green and Brock, 2000). Research has demonstrated that emotional engagement, empathy, and narrative immersion are potent predictors of the appreciation of TV dramas (Lin, 1993), with narrative transportation being a critical component of the emotional connection between international fans and transnational TV dramas (Kim et al., 2019). Koopman (2015) suggested that an individual’s affective orientation toward characters affects their fondness for the story. As for the model developed by this study, it primarily focuses on assessing how AD (including identifying with characters, adopting their understanding of narrative events, and sharing emotions) influences transportation. Consequently, we formulate the following hypothesis:

*Hypothesis 3:* Affective disposition is positively associated with transportation.

### 2.5 Cultural identity as a moderating variable between social norms and transportation

To better understand the impact of norms, it is necessary to consider a variety of moderating factors (e.g., Chung and Rimal, 2016; Kryston and Eden, 2022; Carstens, 2003). Tan’s (2021) NECT model (Narrative Effects for Cultural Targeting) confirmed that cultural similarity positively moderates perceived SN and narratives. In the E-ELM model (Slater and Rouner, 2002; Tan, 2021), the EORM model (Moyer-Guse, 2008; Tan, 2021), and the NECT model (Tan, 2021), culture is considered a moderating variable between SN and narratives. Larkey and Hecht (2010) highlights that socio-cultural variable, such as attitudes, beliefs, and behaviors, influence the narrative process and outcomes. Narratives should be rooted in culture, with culture acting as a specific norm and mediator in storytelling (Tan, 2021).

Researchers have largely examined individuals’ interactions with media content as a way to determine the impact of social factors on media enjoyment (Perse, 2000; Kryston and Eden, 2022). Tal-Or and Cohen (2010) believed that considering these patterns of connectivity in different cultural contexts will significantly impact how we conceive of CI and media enjoyment. One of the most apparent manifestations is that people are exposed to foreign media while maintaining a solid connection to their cultural heritage (Hall, 2003). Subsequent research has revealed that, at the individual level, people tend to be more attracted to characters and narratives that reflect their cultural background. This is because the socio-environmental elements of audiences can also have a distinct and regulating impact on their enjoyment and preferences (Tiede and Appel, 2020). Media research has investigated the direct correlation between the intensity of one’s identification and their choices for entertainment (Trepte, 2006). However, researchers who study norms have contended that the influence of group CI and context on behavior is only moderate (Chung and Rimal, 2016). The CI of individuals can significantly impact how they perceive and respond to cross-cultural media consumption, which in turn affects the degree to which they engage with the narrative (Tal-Or and Cohen, 2010; Tamborini and Weber, 2020). Zhao and Liu (2018) found that CI plays a significant role in audience acceptance of characters in international TV dramas, their love for them, and their involvement in the drama’s storyline. Subsequently, we frame the following hypothesis:

*Hypothesis 4:* Cultural identity moderates the relationship between social norms and transportation.

### 2.6 Enjoyment and transportation

Enjoyment, defined as a pleasurable emotional response to media consumption (Raney, 2003; Andrejevic and Volcic, 2020), is one of the key factors motivating audiences to continue watching a program (Zillmann and Bryant, 1994; Bilandzic and Busselle, 2011; Bryant et al., 1981; Kukkakorpi and Pantti, 2021; Valkenburg and Cantor, 2000). Media enjoyment is typically considered an overall cheerful disposition and a liking for media content (Tribusean, 2020; Vorderer, 2003). Nabi and Krcmar (2004) developed a model that establishes three dimensions of pleasure: cognitive, affective, and behavioral, which influence each other (Lu et al., 2019). Several studies regarding transportation have concluded that there is a strong correlation between transportation and enjoyment (e.g., Busselle and Bilandzic, 2006; Green et al., 2004; Owen and Riggs, 2012; Grigore and Hoeken, 2016; Koo, 2019). Deng et al. (2022) conducted a meta-analysis testing the expanded transportation model and its antecedents and consequences. The study’s findings suggest that narrative transportation increases enjoyment, attitudes, and behavioral intentions.

Research has extensively examined the enjoyment of various media genres (e.g., Nabi and Krcmar, 2004), violent entertainment (e.g., Aubrey et al., 2012; Lin and Xu, 2017; Thompson et al., 2021), emotional films (Billieux, 2019; Thompson et al., 2021), and online TV dramas (Flayelle et al., 2019; Dey et al., 2020; Sánchez Laws, 2020). Furthermore, numerous studies have examined the influence of transportation theory on audiences’ satisfaction with international TV dramas (Lu et al., 2019). Specifically, audiences who experienced more transportation reported a greater appreciation for the TV dramas (Cohen et al., 2016; Raney, 2013). Kim et al. (2018) conducted a study that revealed that plot twists in Korean dramas caused a surge in audience engagement and enjoyment. This implies that unforeseen narrative changes can positively influence the story’s impact and the audience’s engagement (Walker, 2023). Some research has focused on investigating how narrative transmission and cross-cultural empathy influence the perception of Korean dramas by audiences from other cultures (Hoffman, 2000; Shin, 2019; Kim et al., 2021). The researchers discovered that narrative transfer connected cross-cultural empathy and audience enjoyment, indicating that transfer theory is an essential framework.

This study suggests that the SN and AD of the audience when watching transnational dramas indirectly influence their transportation experience and the satisfaction, they derive from it. Additionally, CI acts as a moderator between SN and transportation. Consequently, we formulate the following hypothesis:

*Hypothesis 5:* Transportation is positively associated with enjoyment.

### 2.7 Transportation as a mediator variable between affective disposition and enjoyment

Prior research has provided empirical support indicating that individuals with higher levels of trait empathy exhibit a stronger inclination toward narrative interaction (Oliver and Bartsch, 2010). Furthermore, studies have shown that cultural factors, such as knowledge of narrative backgrounds and CI, significantly influence transportation (Cohen and Shrum, 2001). However, despite evidence of the efficacy of these and other similar constructs, a deeper understanding of their function within narrative experiences, the connections between them, and their influence on persuasion and reality construction is still necessary. The theoretical foundation of mental imagery transportation (Green and Brock, 2002) is challenging to articulate within the context of TV dramas or films. Many scholars have noted the difficulty in separating character identification from the environments and situations in which characters are placed (Cohen, 2001; Eden and Tamborini, 2017; Grizzard et al., 2021; Tamborini and Weber, 2020; Grizzard et al., 2020; Tamborini et al., 2021a, 2021b).

Many existing theories hold that audience character recognition is based on similar preferences. However, little is known about the reasons and methods for audience character recognition (Walkington et al., 2020). Mainstream theories contend that similarity and personal preferences play an important role in determining feelings of identification (Black et al., 2019; Walkington et al., 2020). It is necessary to clarify the connection between cultural elements that influence common SN within a group and the degree of participation in the narrative world when watching global dramas. To understand the fundamental principles of transportation theory more deeply, it is essential to grasp the interplay between specific story factors and specific audience factors in assessing media enjoyment and the interactions among these factors (Thompson et al., 2021). Conducting studies based on the transportation theory model is crucial for predicting and elucidating narrative engagement and media enjoyment for audiences, especially regarding transnational TV dramas. Therefore, we formulate the following hypothesis:

*Hypothesis 6:* Transportation mediates the relationship between affective disposition and enjoyment.

### 2.8 Transportation as a mediator variable between social norms and enjoyment

As the theory of transportation has demonstrated, when the audience enters the narrative, it needs to connect with the characters’ emotions and cognitions (Green, 2004). This connection can be achieved through quasi-social interaction or identification with the characters in the narrative (Mou and Shen, 2018; Cohen, 2001; Green, 2004; Owen and Riggs, 2012). Therefore, connecting with characters plays a vital role in enjoying a narrative. Similarly, pursuing pleasure encourages the audience to continue immersing themselves in the narrative. Watching TV dramas, movies, and other entertainment products is primarily motivated by the desire for hedonistic experiences (Oliver, 2003; Klimmt and Vorderer, 2003; Jacko, 2009; Owen, 2007). Consequently, enjoyment is considered the resulting factor in our study, with transportation acting as the mediating variable. In light of this, we propose the following hypothesis:

*Hypothesis 7:* Transportation mediates the relationship between social norms and enjoyment.

### 2.9 The framework of the research

Based on available research, studies on Malaysian youth’s interest in foreign TV dramas have primarily focused on evaluating their visual impact (Modili et al., 2022; Ching et al., 2020; Lee et al., 2020; Hashim, 2019) and addictive behaviors (Nik Jaafar et al., 2021; Jie Chan et al., 2022). These studies particularly focus on the narrative structure and the aspects that contribute to the enjoyment of these dramas. However, there has been little research on the influence of international TV dramas on media engagement and increased enjoyment. Accordingly, this study proposes an extended transportation theory based on the transportation theoretical framework, including perceptual normative models and cultural examination factors from EORM and NECT, to assess narrative immersion and enjoyment among Malaysian youth while watching transnational TV dramas.

**Figure 1:**
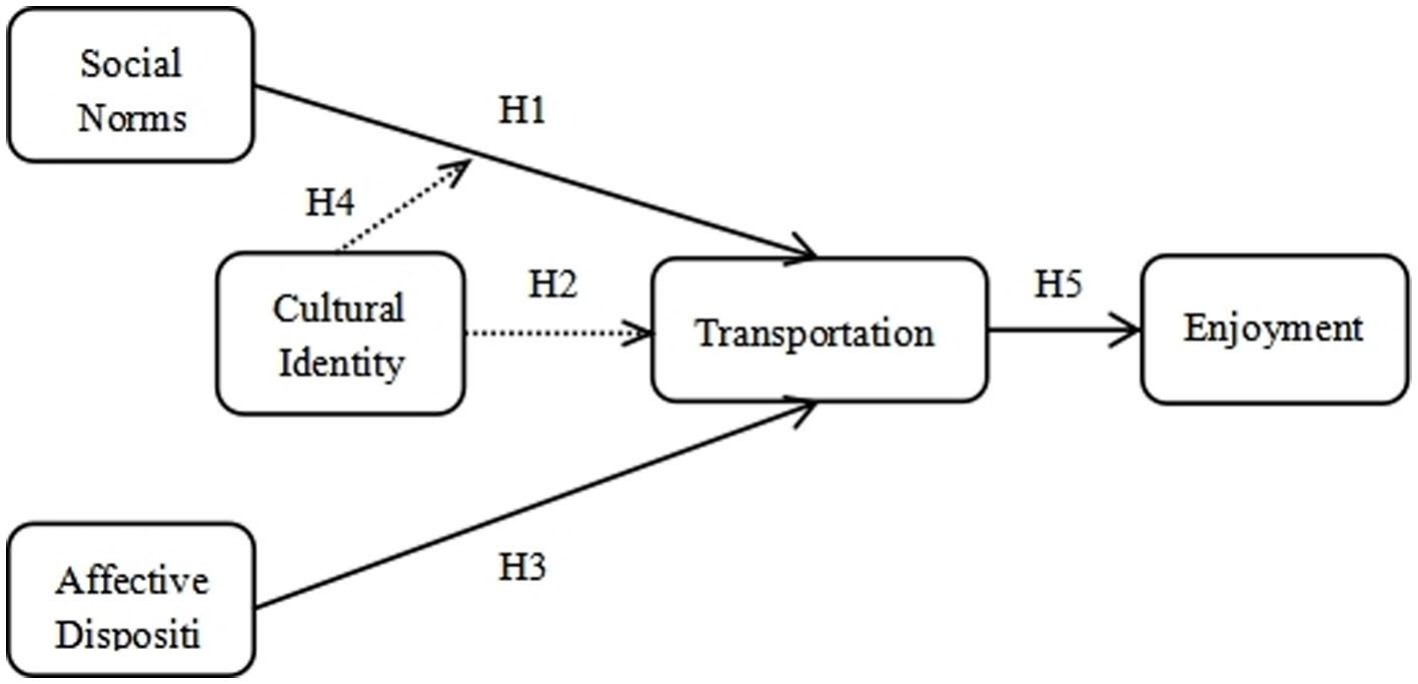
Theoretical model.

The relationships to be analyzed in the framework (Figure 1) of this study are: (1) the influence of SN perceived by the audience on transportation; (2) the relationship between audiences’ AD and the influence of transportation; (3) the possible moderating effect of CI on SN and transportation; (4) CI may have a direct influence on transportation; (5) the effect of transportation on enjoyment; and (6) transportation plays a mediating function between AD and media enjoyment, as well as between SN and media enjoyment.

The current research attempts to augment the body of transportation research through these relatively innovative aspects: (1) It employs structural equation modeling (SEM) instead of regression analysis to examine SN, AD, transportation, and enjoyment; and (2) The model incorporates CI as a variable moderating the influence of SN on transportation.

## 3 Methods

### 3.1 Research approach and sampling

Given the challenge of sampling the entire population due to its size, we adopted a purposive sampling method. The study recruited a cohort of 400 participants, exclusively comprising students from a public university in Malaysia. This cohort included students from both a comprehensive general education cinema course and an advanced communication studies course. Questionnaires were distributed along with informed consent forms. To encourage genuine responses, additional academic credits were provided as an incentive. Participants dedicated approximately 2.5 h to viewing the Chinese TV drama “Story of Yanxi Palace” (2018), which consisted of 3 episodes, and subsequently filled out a written questionnaire.

### 3.2 Measurements and procedure

We adapted the questionnaires used for this study from established literature, including the Audience Response Scale (Oliver and Bartsch, 2010), the Transportation Scale Short Form (Appel et al., 2015), the Descriptive Norms and Prohibitive Norms Scale (Park and Smith, 2007), the Psychological Assessment of Multiracial CI Scale (Felix-Ortiz et al., 1994), and the Addiction Scale (AD Scale) (Owen and Riggs, 2012). After conducting a pre-test and making necessary adjustments, we produced the final questionnaire.

This study investigates SN primarily through audiences’ perceptions of societal norms, using the descriptive norm scale by Park and Smith (2007) (e.g., “My close family members and friends all recommend watching Chinese TV dramas”). The measurement of AD is adapted from the Liking of Leonard Scale by Owen and Riggs (2012), assessing audiences’ affective orientations through scales measuring preferences for characters and TV show content (e.g., “I always feel that I resemble one of the main characters in Chinese TV dramas”). The short Transportation Scale by Appel et al. (2015) is used to measure whether audiences are fully transported into the narrative world or immersed in the narrative experience (e.g., “While watching, I am mentally involved in the story”). The measurement of CI is derived from the Multiethnic Identity Measure (MEIM) developed by Phinney (1990), utilizing the subscale that measures the degree of identification with other ethnic or cultural characteristics (e.g., “I’m familiar with Chinese history and politics”), examining dimensions of art, history, and language. Enjoyment is measured using the three-item enjoyment scale by Oliver and Bartsch (2010), which includes dimensions of fun, meaning, lasting impression, and suspense (e.g., “I like Chinese TV dramas very much”). Higher scores indicate greater enjoyment. The questionnaire adopted a five-point Likert scale ranging from 1 (indicating strong disagreement) to 5 (indicating strong agreement).

To observe more accurate audience responses to transportation and enjoyment, we referenced the testing method by Owen and Riggs (2012), which presents a medium level of cognitive challenge through a unique narrative strategy: narrating in reverse order from the main storyline. Specifically, we re-edited the TV drama to present each subsequent scene earlier in the story timeline than the previous one (Owen and Riggs, 2012). This method allows audiences to move backwards through the scenes to the start of the story. This is because the psychological representation of textual meaning, as part of the comprehension process, generates relevant narrative understandings through flow reasoning (McNamara and Magliano, 2009). Since flow implies that narrative engagement is automatic (Busselle and Bilandzic, 2009), the current study aims to use cognitively challenging narrative content to examine whether audiences can still be transported into the narrative world and derive media enjoyment, despite reduced coherent flow situations.

Csikszentmihalyi (1990) explained that flow occurs when there is a good match between the participant’s capabilities (Owen and Riggs, 2012) or inclinations and the challenges of the activity. Thus, reducing flow requires narratives that are sufficiently challenging and interesting but not to the extent that they become incomprehensible, which would be optimal (Owen and Riggs, 2012). If the cognitive challenge presented by the narrative is too great, audiences will not understand, become frustrated, and the transportation experience will cease. A weak cognitive challenge intensity will cause the audience to become bored and will pull them out of the story world. It has been shown that temporal order anomalies in narratives increase cognitive load during the process of narrative comprehension (Zwaan, 1996; Owen, 2007; Owen and Riggs, 2012). The importance of sequencing in narrative understanding for transportation is reflected in its frequent manipulation in empirical studies in discourse psychology (Graesser et al., 2002; Radvansky et al., 1998; Rinck et al., 2001; Owen, 2007). According to constructivist theory, reverse sequencing can increase the cognitive challenge for audiences by reducing coherent mental situational models, making the narrative harder to understand compared to the original, thus increasing the cognitive challenge (Owen and Riggs, 2012). Consequently, we decided to reverse the TV drama to test whether audiences could still engage in narrative immersion and enjoy the media experience. Additionally, we need to examine the mechanisms by which SN, CI, and AD influence audiences’ narrative immersion in disrupted flow situations.

### 3.3 Procedure

We chose the Chinese TV drama “Story of Yanxi Palace” (2018) because it possesses highly recognizable Chinese cultural features, particularly as a distinct genre—historical palace drama. We employed a unique narrative strategy by re-editing the drama to present the main storyline in reverse chronological order, where each subsequent scene occurs earlier in the story timeline. The plot of “Story of Yanxi Palace” can be summarized as follows: A young girl named Wei Yingluo enters the palace as a maidservant to uncover the truth behind her sister’s death. The drama consists of two intertwined yet independent plot lines. The colored sequences (where most of the story unfolds) depict Wei Yingluo’s journey as she becomes a maidservant in the palace and attempts to find concrete evidence of her sister’s murder. The black-and-white sequences are flashbacks of events leading up to her sister’s death, as well as her memories.

We used a reverse chronological design to manipulate the story’s timeline in “Story of Yanxi Palace” to disrupt the audience’s attempt to establish coherence in several dimensions: the causal relationships of actions, story spatiality, and character motivations. We re-edited the film’s original temporal structure to make it difficult for viewers to determine causal relationships by: (1) having the colored sequences (which account for most of the film) move backward in time, with each subsequent scene occurring at an earlier point than the previous one; and (2) having the black-and-white sequences move forward in time, breaking the logical continuity with the original colored sequences. Due to this highly original narrative structure, viewers are forced to rethink causal relationships and attempt to re-establish related facts almost after every scene.

From April to May 2023, the researchers conducted a study on undergraduates at a public university in Malaysia. Participants gathered in groups of 15 to 40 in a lecture hall. Before the drama was shown, researchers read out a study briefing to ensure participants understood the study’s intent and followed the instructions. They were told they could leave the screening room at any time without penalty. Participants then signed informed consent forms. The hall lights were turned off, and participants watched the edited version of “Story of Yanxi Palace.” After the TV drama ended and the lecture hall lights were turned back on, participants immediately filled out a questionnaire that included items on SN, preferences for the protagonist or story, CI, enjoyment, and transportation.

## 4 Results

### 4.1 Validating the measurement models: confirmatory factor analysis (CFA) model

After a comprehensive screening of the data, we identified 353 valid responses that were utilized for our study, as shown in Table 1. The model parameters were estimated using IBM SPSS AMOS 24.0 software, employing the maximum likelihood estimation technique. Wu (2018) stated that structural equation modeling (SEM) analysis usually starts with confirmatory factor analysis (CFA) (Juan et al., 2022) to ensure that the measurement models for the underlying structures are correct based on theoretical foundations.

**Table 1:** Demographics of respondents (*N* = 353).

Characteristic	M	Percent (%)
**Gender**		
Male	201	56.94
Female	152	43.06
Age		
≤19	61	17.28
20–21	94	28.48
22–23	142	40.23
≥24	56	15.86
**Education**		
Matriculation/Pre-University	95	26.91
Bachelor’s degree	229	64.87
Master	29	8.21
**Ethnic**		
Malay	264	74.78
Chinese	32	9.06
Indian or others	57	16.14

Consequently, CFA effectively assesses the validity and reliability of research constructs. As shown in Table 2, CFA results are presented from the perspective of standardized factor loadings (SFL), composite reliability (CR), and average variance extracted (AVE) (Juan et al., 2022). The correlation coefficients of the various aspects of the questionnaire are presented in Table 3. For all the constructs in this study, the standardized factor loading (SFL) is over 0.50 on average, and the composite reliability (CR) is between 0.800 and 0.896. These figures come from a measurement model with five constructs, which were checked using CFA. Some values are above the acceptable threshold of 0.70 (Hair Jr et al., 2020), indicating strong internal consistency and convergent validity of the constructs involved. Additionally, the range of AVE values is between 0.502 and 0.742, which is higher than the minimum critical value of 0.5 suggested by Shevlin and Miles (1998), providing further proof of convergent validity.

**Table 2:** SFL, CR, AVE of items of the academic dishonesty questionnaire.

Constructs	SFL	CR	AVE
1-Social Norms (SN) (Park and Smith, 2007)		0.847	0.649
SN1: My close family members and friends all recommend watching Chinese TV dramas.	0.773		
SN2: My close family members and friends all agree that I should watch Chinese TV dramas.	0.74		
SN3: My close family members and friends prefer to watch Chinese TV shows.	0.896		
2-Affective Dispositions (AD) (Owen and Riggs, 2012)		0.896	0.742
AD1: I want to be the main character in my favorite Chinese TV dramas.	0.835		
AD2: I always feel that I resemble one of the main characters in Chinese TV dramas.	0.898		
AD3: I feel that I feel and know the world in the same way as the main character.	0.85		
3-Transportation (T) (Appel et al., 2015)		0.895	0.682
T1: While watching, I am mentally involved in the story.	0.792		
T2: While watching, I was completely immersed in the story.	0.879		
T3: I am impressed by the main clauses of Chinese TV dramas I have watched.	0.815		
T4: I am often emotionally affected by films and TV dramas.			
4-Cultural Identity (CI) (Phinney, 1990)		0.800	0.502
CI1: I’m familiar with Chinese painting and other arts.	0.683		
CI2: I’m familiar with Chinese history and politics.	0.788		
CI3: I’m familiar with Chinese legends and symbols.	0.642		
CI4: I’m able to speak and understand Chinese.	0.713		
5-Enjoyment (E) (Oliver and Bartsch, 2010)		0.846	0.648
E1: I like Chinese TV dramas very much.	0.753		
E2: If I had the chance, I would watch my favorite Chinese TV dramas again.	0.831		
E3: I would recommend my friends to watch Chinese TV dramas.	0.829		

SFL, standardized factor loadings; CR, composite reliability; AVE, average variance extracted.

**Table 3:** Correlations between variables.

Constructs	1	2	3	4	5
1.SN	0.806				
2.AD	0.470^**^	0.861			
3.T	0.563^**^	0.417^**^	0.826		
4.CI	0.354^**^	0.215^**^	0.615^**^	0.709	
5.E	0.398^**^	0.286^**^	0.585^**^	0.368^**^	0.805

Squared root of AVE values are noted on the diagonal with bold. ^*^Correlation is significant at the 0.05 level. ^**^Correlation is significant at the 0.01 level (two-tailed).

Moreover, we found that the correlations between different constructs (expressed as non-diagonal values) are lower than the square root of the average variance extracted (AVE) (expressed as diagonal values). According to Baliao (2023), this provides strong evidence of discriminant validity. The CFA analysis demonstrated that both the obtained data and the proposed CFA measurement models were entirely appropriate (Akhtar et al., 2024; Juan et al., 2022). To validate the research hypotheses of this study, the next step is to assess the fit indices of the proposed structural models (Juan et al., 2022) to determine their validity.

### 4.2 Analyzing the structural model

**Figure 2:**
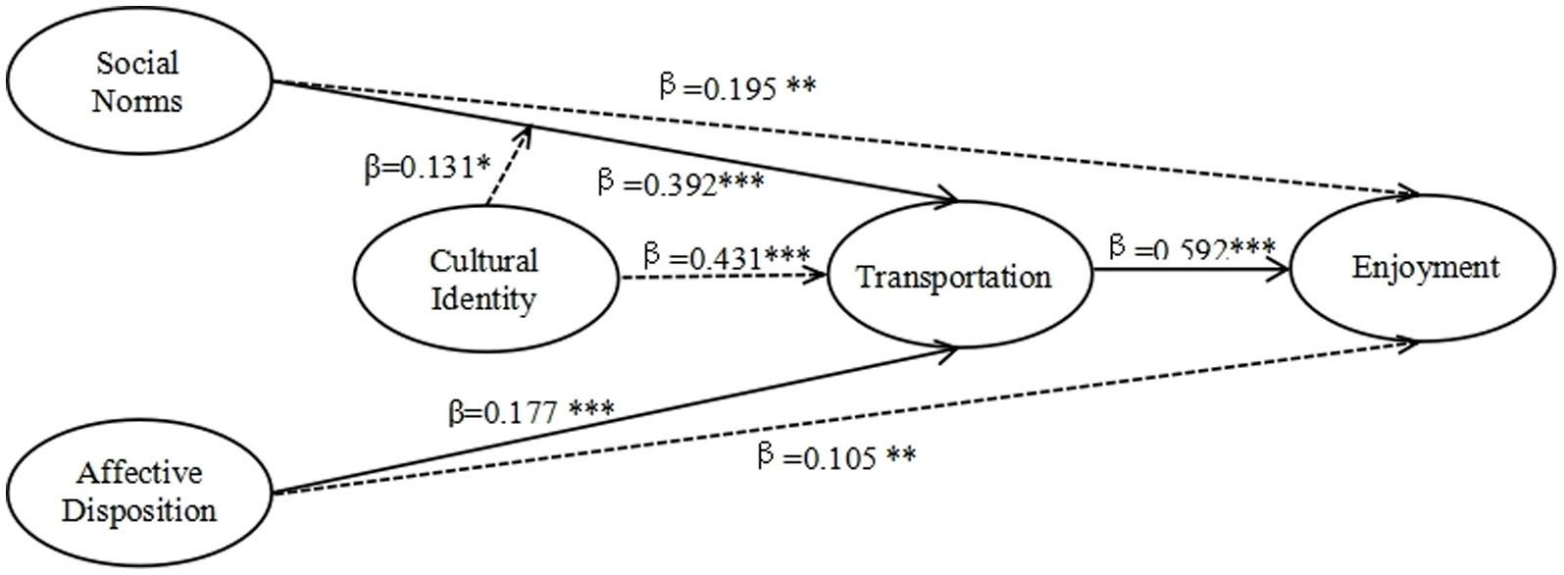
Results of structural equation model (^*^p = 0.05. ^**^p = 0.01. ^***^p = 0.001).

The assessment of model adequacy by the structural model is crucial to ensure the achievement of three categories of fit indices. The pertinent indicators for evaluating fit include: (1) Absolute fit: Chi-Square and Root Mean Square Error of Approximation (RMSEA); (2) Incremental fit: Goodness of Fit Index (GFI), Normed Fit Index (NFI), Tucker-Lewis Index (TLI), and Comparative Fit Index (CFI); and (3) Parsimonious fit: Chi-Square/df (Ghouri et al., 2024; Zhenjing et al., 2022). Figure 2 displays the model fit statistics.

Table 4 shows that all three types of model fit indices—absolute, incremental, and parsimonious—indicate that the four proposed models fit well. The researchers adopted the suggested structural model for subsequent hypothesis testing.

**Table 4:** Fit statistics for model.

Index	*χ*^2^	*χ*^2^/df	GFI	RMSEA	NFI	TLI	CFI
Result	160.23	1.470	0.948	0.037	0.952	0.980	0.984

### 4.3 Testing the hypotheses

Based on the verification of the results of the measurement constructs with the current data set, the next step in this study is to test the hypotheses using structural equation modeling (SEM). For this purpose, we used IBM SPSS AMOS 24.0 software and non-standardized estimates, along with regression weight findings, to test the research hypotheses.

**Figure 3:**
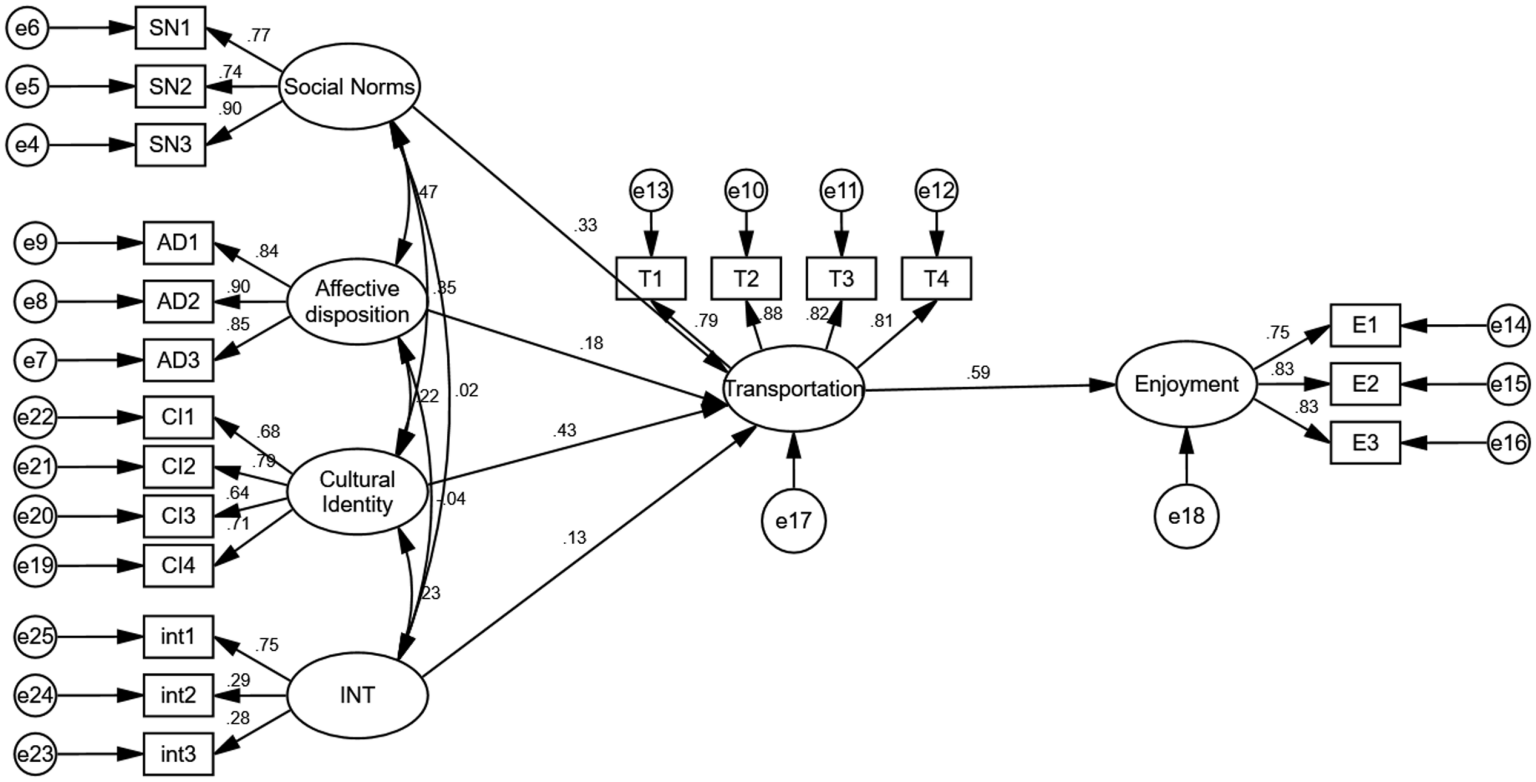
Fit statistics for model.

Table 5 presents the unstandardized regression coefficients from the path analysis of this study, which aims to test the significance of the relationships between the variables and evaluate the degree of support for the hypotheses according to the collected data. Based on the structural equation model, Figure 3 shows the standardized estimates for the proposed model.

**Table 5:** Unstandardized path coefficients to testing the causal effects of the constructs for model.

Hypotheses	Construct	Path	Construct	Estimate	S.E.	C.R.	*p*	Result
H1	T	<---	SN	0.296	0.052	5.678	^***^	Support
H2	T	<---	CI	0.591	0.084	7.067	^***^	Support
H3	T	<---	AD	0.161	0.048	3.354	^***^	Support
H4	T	<---	SN^*^CI	0.391	0.195	2	0.045	Support
H5	E	<---	T	0.491	0.051	9.698	^***^	Support

From Table 5, it is evident that the contributions of all constructs to the endogenous constructs are of critical importance. There are five paths covered by the model of this study, and each of them has statistical significance (*t*-value >1.96, *p* < 0.001). Hence, the findings from this study conducted at a Malaysian public university support the five hypotheses under investigation. Specifically, SN (*β* = 0.329, p < 0.001), CI (β = 0.431, *p* < 0.001), AD (β = 0.177, *p* < 0.001), and SN^*^CI (β = 0.131, *p* < 0.05) exhibit significant associations with T and collectively account for 43.3% of the variance explained. Moreover, Transportation (β = 0.592, *p* < 0.001) significantly influences Enjoyment, and together, they explain 35.7% of the variance observed in the study sample.

### 4.4 Testing the mediating effects

To further investigate the mechanisms through which SN and AD influence the enjoyment of media by local audiences, we must explore the indirect effects enabled by the mediating variable, Transportation. Table 6 provides a summary of the results from the data.

**Table 6:** Bootstrap test for significance of mediating effects.

Hypotheses	Path	Estimate	Boot SE	Bias-corrected 95%CI		*p*	Result
				Lower	Upper		
H6	SN → T → E	0.195	0.044	0.117	0.285	0.001	Support
H7	AD → T → E	0.105	0.037	0.035	0.179	0.005	Support

The significance of the mediation effect was evaluated using the indirect effect/standard error (SE) of 1.96 or the presence of a non-zero value in the confidence intervals of the percentile approach and the bias-corrected procedure (Kohn and Smith, 2003; Ledermann and Macho, 2009; Jiao et al., 2024). Generally, partial mediation occurs when the direct effects remain significant, while complete mediation occurs when the direct influence is no longer significant (Jun et al., 2023). The current study revealed a notable mediation effect between SN and enjoyment through the transportation variable. The coefficient for this influence was 0.195, and the 95% confidence interval ranged from 0.117 to 0.285, excluding zero. One’s AD substantially influences the experience of enjoyment through the transportation process, as indicated by the results presented in Table 6. The computed mediation effect was 0.105, with a 95% confidence interval of (0.035, 0.179), which does not include zero. The findings of this study indicate that mediation in modes of transportation, resulting from alterations in SN and AD, leads to modifications in the sense of enjoyment. The indirect effects are not independent but manifest through transportation.

### 4.5 Testing the moderating effect

The study considered the moderating role of CI while examining the association between transportation (a dependent variable) and SN (an independent variable). Using the statistical program SPSS, a model was adopted to examine this moderating influence. Transportation, SN, and CI were all normalized independently before analysis. An interaction term (SN ^*^ CI) was generated to capture the interaction between the independent variable SN and the moderator variable CI.

**Figure 4:**
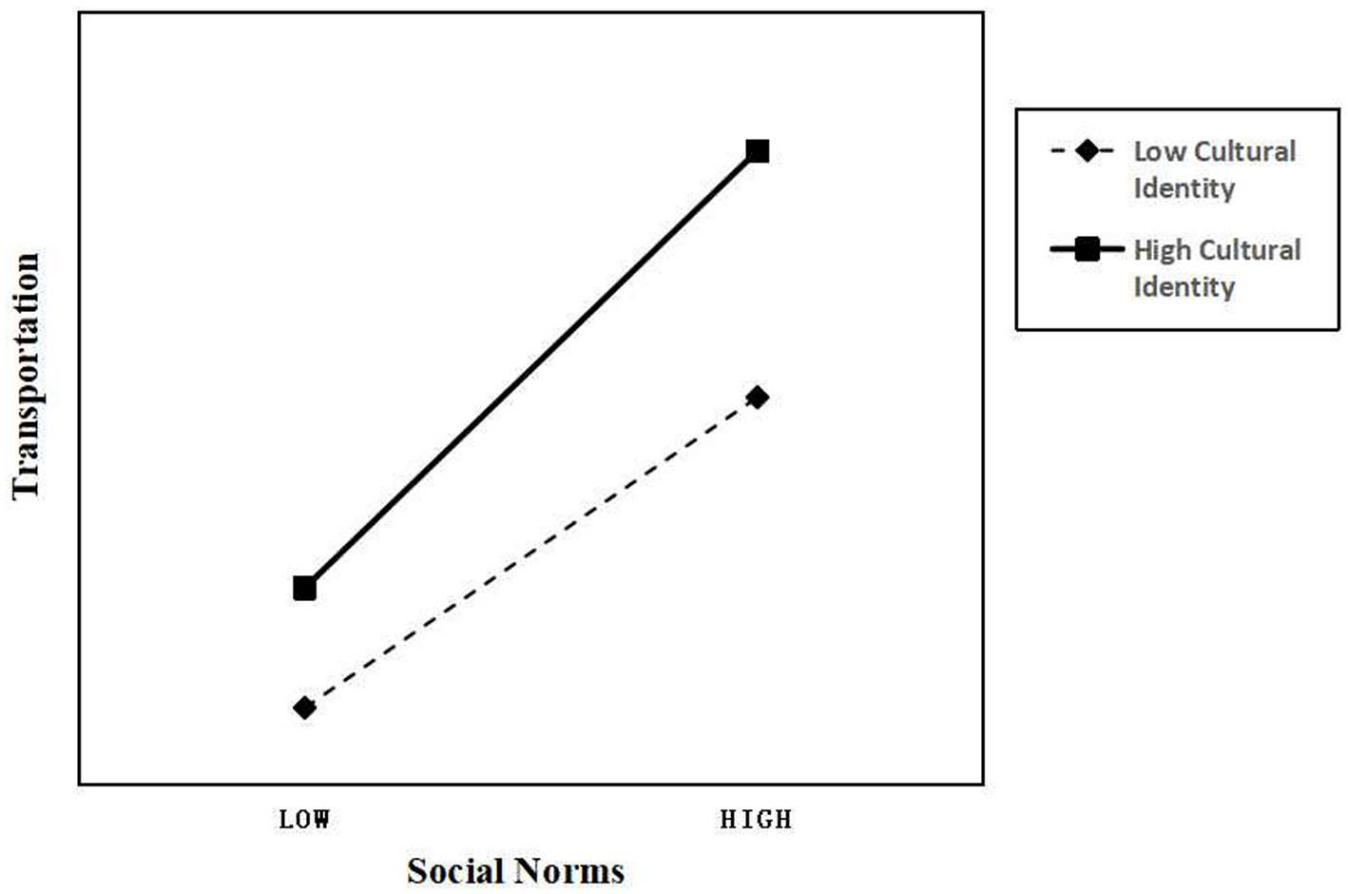
Moderating effect.

The results shown in Table 7 and Figure 2 indicate that the correlation between transportation and CI is 0.4749. A *p*-value of less than 0.05 indicates the statistical significance of this coefficient (Jia et al., 2024), confirming that CI has a positive and statistically significant effect on transportation. The current analysis indicated that, at a significance level of *p* < 0.05, the coefficient of the interaction term SN ^*^ CI on transportation was 0.1639. This result implies the presence of a sizable moderating influence, suggesting that the relationship between SN and transportation is positively moderated by CI (Figure 4).

**Table 7:** Moderating effects testing.

	Effect	S.E.	*t*-value	*p*	LLCI	ULCI
constant	3.6004	0.0361	99.8659	0	3.5295	3.6713
SN	0.4023	0.0427	9.4237	0	0.3183	0.4862
CI	0.4749	0.053	8.9635	0	0.3707	0.5791
SN^*^CI	0.1639	0.0677	2.4213	0.016	0.0308	0.2971

## 5 Discussion

This study examined how audience perceptions of SN, CI, and AD explain the mechanisms of narrative immersion and enjoyment in a cross-cultural context. The results revealed that SN, CI, and AD significantly influence transportation. Transportation served as a mediator in the relationship between SN’s influence on enjoyment and AD’s influence on enjoyment. This study provides compelling evidence supporting the statistically significant effect of SN on the moderating influence of transportation under CI. The present research project was conducted within a comprehensive conceptual framework. The test of the path model validates both the proposed conceptual framework and the research hypotheses.

This study revealed that SN significantly and positively influences transportation and indirectly influences enjoyment. This influence is indirect rather than occurring in isolation, which is consistent with our hypotheses. SN is a strong predictor of both transportation and enjoyment, aligning with previous research suggesting that societal norms/influences (e.g., recommendations from friends, opinions from family) affect audience narrative cognition and emotional immersion in foreign TV dramas (Trepte and Loy, 2017; Park et al., 2020; Weaver and Frampton, 2019; Kryston and Eden, 2022). SN, as unwritten codes of behavior deemed acceptable by society, play a pivotal role in moderating the narrative transportation process. Storytelling that complies with SN while offering new perspectives can maximally engage audiences, potentially altering their narrative cognition and deepening their immersion in fictional narratives (Green, 2008; Green, 2004).

According to the results, CI significantly and positively influences enjoyment through transportation, which aligns with the studies conducted by Straubhaar (1991), Moyer-Guse (2008), and Tan (2021). This finding indicates that, in a cross-cultural context, culture is a crucial resource that audiences utilize to understand media products and enhance their enjoyment through narrative immersion. In addition, CI, as a moderating variable, significantly promotes the positive relationship between the social paradigm of audience perception and immersion in media narrative content. Consequently, this study confirms the importance of CI and demonstrates how cultural values reinforce and facilitate the impact of perceived SN on narrative immersion. Immersing oneself in a narrative world is a multifaceted process involving cognitive (Owen and Riggs, 2012; Bozeman, 2022), emotional, and imaginative engagement with the story (Straubhaar, 1991). Furthermore, the findings of this research offer a fresh psychological perspective on how cultural distance enhances enjoyment when appreciating transnational media content (Baek and Kim, 2016). On one hand, viewers who identify strongly with a certain culture may become more accustomed to the cultures of other nations and find it easier to comprehend the plots of foreign TV dramas. Local viewers, who can relate to the characters or themes through shared cultural values and historical ties, are more likely to become engrossed in the story. On the other hand, the “novelty” of foreign TV series can enhance audience delight, which explains why cultural distance the opposite effect by can have increasing enjoyment. CI can facilitate this process by providing familiar frameworks, themes, and cultural cues that resonate with the audience’s experiences and values. In the context of cross-cultural media content acceptance, the moderating effect of CI aligns with previous research by Cohen (2001) and Tamborini and Weber (2020), suggesting that CI can shape audience perceptions and reactions to cross-cultural media consumption, ultimately influencing the degree to which audiences are drawn into the narrative. Therefore, CI acts as a mechanism to transform SN into deeper narrative immersion.

AD has a positive influence on transportation. This is consistent with studies conducted by Green and Brock (2000), Owen and Riggs (2012), Shafer and Raney (2012), Kim et al. (2019), Zibrek et al. (2018), MacDorman (2019), Lu (2020), and Thompson et al. (2021). This indicates that the higher the transportation in TV dramas, the greater the narrative immersion experience when the personal emotional experiences of Malaysian audiences align with the characters, situations, or emotional experiences in Chinese TV dramas. Moreover, AD is an essential predictor of transportation, consistent with previous research by Paluck (2009), Walkington et al. (2020), Grizzard et al. (2023), and Nicolls et al. (2024), where emotional connection to characters in the narrative or to situations in the drama significantly influences the overall cognitive and immersion levels in the narrative (Cohen and Klimmt, 2021). Narrative immersion theory posits that well-structured narratives can ‘transport’ audiences into the world of the story, fostering empathy, reducing counter-arguing, and cultivating positive attitudes towards the narrative (Raney, 2003; Zillmann and Cantor, 1976; Campbell et al., 2020). In a media culture atmosphere aimed at enjoyment, whether through popular characters or story situations, they are more likely to induce narrative immersion in the audience and thus increase media enjoyment (Zibrek et al., 2018).

Notably, AD significantly and indirectly influences enjoyment, primarily through the transportation mechanism, consistent with previous research by Coplan (2004), Kupferberg et al. (2011), Kang et al. (2020), and Nicolls et al. (2024). However, it is essential to emphasize that AD do not have a direct residual influence on enjoyment. This means that, while the audience’s liking for the protagonist does not produce enjoyment without transportation, a higher level of alignment between the protagonist and transportation elements ultimately leads to greater media enjoyment and satisfaction. Furthermore, it is worth pointing out that one of the controlling factors in this study is the use of flashbacks to convey story information. Even though such an approach might disrupt participants’ experience of flow, our findings conflict with Owen and Riggs’ (2012) conclusions, as there needs to be evidence that audiences perceive a more significant challenge in the narrative. As a result of the moderating effects of culture, strong cultural symbols can lead audiences to put in more effort to understand the non-linear narrative structure, potentially reducing transportation obstacles.

## 6 Limitations and future research

Researchers should consider the aforementioned findings within the confines of this study’s constraints, which also suggest potential avenues for future research. Initially, the breadth of the study’s sample is confined to mainland Chinese TV dramas, limiting the ability to extrapolate the findings. While mainland Chinese TV dramas have significant popularity in Asia, rivaling similar media products from countries like Japan and South Korea, they nevertheless fall behind in terms of global recognition. Given this context, conducting a comprehensive comparison of media enjoyment among audiences in different countries could yield more universal results based on an in-depth analysis of audiences’ media consumption habits.

However, the current research focuses solely on quantitative and cross-sectional analyses, which undoubtedly have limitations. Future investigations should consider incorporating a qualitative component to delve deeper into audiences’ narrative immersion and enjoyment behaviors toward international TV dramas. Furthermore, further studies should examine the diverse forms of foreign media content consumption behaviors that have emerged in contemporary times. Specifically, in the post-pandemic period, it is important to consider the various ways in which people consume foreign media content through online and networked technologies. This includes focusing on the distribution of shared content online and understanding the psychological processes through which audiences share their narrative experiences.

## 7 Conclusion

This study explores Malaysian audiences’ engagement with the narrative world and their enjoyment when watching complex and exciting Chinese TV dramas. Among Malaysian audiences, SN, CI, and AD play essential roles in influencing the complex and subtle correlation between engagement and enjoyment within the narrative world. As discussed in this study, media enjoyment extends beyond mere entertainment, delving into audiences’ deeper emotional, conscious, and cultural dimensions. Thus, transnational media enjoyment transcends simple entertainment, encompassing profound emotional, social, and cultural aspects.

In previous studies, different aspects of this association have been emphasized; however, this study offers a new perspective on the discipline by examining a unique form of narrative immersion within a transnational context. By understanding these correlations, we can explore future theoretical research and practical applications in TV drama content production, cultural exchange, and the analysis of global media. The framework model examined in this study combines SN, CI, AD, and an individual’s participation in stories into a cohesive structure that can be applied across cultures. This framework model provides valuable insights into human storytelling and media enjoyment. In addition to enhancing the theoretical framework of media psychology, this understanding also offers practical recommendations that artists can utilize to produce meaningful, engaging, and culturally relevant content in the future.

## Data Availability

The raw data supporting the conclusions of this article will be made available by the authors, without undue reservation.
