# Supplementary material for: The influence of transportation, social norms, cultural identity, and affective disposition in transnational media enjoyment
Source: Front Psychol. 2024 Nov 21;15:1377898. doi: 10.3389/fpsyg.2024.1377898 (PMC11619051; doi:10.3389/fpsyg.2024.1377898)
Supplement: Supplementary file 1 [file Data_Sheet_1.docx]

Supplementary Material

# Supplementary Figures and Tables

## Supplementary Figures

H5

H4

H3

H2

H1

Social

Norms

Cultural Identity

Affective

Disposition

Enjoyment

Transportation

**Supplementary Figure 1**. Theoretical model

**Supplementary Figure 2.** Results of structural equation Model.

Note. *p=.05. **p= .01. ***p= .001

β=0.105 **

β=0.177 ***

β=0.431***

β=0.592***

β=0.392***

β=0.131*

β=0.195 **

Social

Norms

Affective Disposition

Cultural

Identity

Transportation

Enjoyment

## Supplementary Tables

| Characteristic | M | Percent (%) |
| --- | --- | --- |
| Gender |  |  |
| Male | 201 | 56.94 |
| Female | 152 | 43.06 |
| Age |  |  |
| ≤19 | 61 | 17.28 |
| 20-21 | 94 | 28.48 |
| 22-23 | 142 | 40.23 |
| ≥24 | 56 | 15.86 |
| Education |  |  |
| Matriculation/Pre-University | 95 | 26.91 |
| Bachelor's degree | 229 | 64.87 |
| Master | 29 | 8.21 |
| Ethnic |  |  |
| Malay | 264 | 74.78 |
| Chinese | 32 | 9.06 |
| Indian or others | 57 | 16.14 |

**Supplementary Table 1**. Demographics of Respondents (N= 353).

| Constructs | SFL | CR | AVE |
| --- | --- | --- | --- |
| 1-Social Norms(SN) （Park & Smith, 2007 ） |  | 0.847 | 0.649 |
| SN1: My close family members and friends all agree that I should watch Chinese TV dramas. | 0.773 |  |  |
| SN2: My close family members and friends all agree that I should watch Chinese TV dramas. | 0.74 |  |  |
| SN3: My close family members and friends prefer to watch Chinese TV shows. | 0.896 |  |  |
| 2-Affective Disposition(AD) （Bradford, 2002 ） |  | 0.896 | 0.742 |
| AD1: I want to be the main character in my favorite Chinese TV dramas. | 0.835 |  |  |
| AD2: I always feel that I resemble one of the main characters in Chinese TV dramas. | 0.898 |  |  |
| AD3: I feel that I feel and know the world in the same way as the main character. | 0.85 |  |  |
| 3-Transportation(T) （Appel, Gnambs, Richter & Green, 2015） |  | 0.895 | 0.682 |
| T1: While watching, I am mentally involved in the story. | 0.792 |  |  |
| T2: While watching, I was completely immersed in the story. | 0.879 |  |  |
| T3: I am impressed by the main clauses of Chinese TV dramas I have watched. | 0.815 |  |  |
| T4: I am often emotionally affected by films and TV dramas. |  |  |  |
| 4-Cultural Identity (CI) （Phinney, 1992） |  | 0.800 | 0.502 |
| CI1: I’m familiar with Chinese painting and other arts. | 0.683 |  |  |
| CI2: Familiarity with Chinese history and politics. | 0.788 |  |  |
| CI3: Familiarity with Chinese legends and symbols. | 0.642 |  |  |
| CI4: I’m able to speak and understand Chinese. | 0.713 |  |  |
| 5-Enjoyment(E) (Oliver & Bartsch, 2010） |  | 0.846 | 0.648 |
| E1: I like Chinese TV dramas very much. | 0.753 |  |  |
| E2: If I had the chance, I would watch my favorite Chinese TV dramas again. | 0.831 |  |  |
| E3: I would recommend my friends to watch Chinese TV dramas. | 0.829 |  |  |

**Supplementary Table 2**. SFL, CR, AVE of Items of the Academic Dishonesty Questionnaire.

| Constructs | 1 | 2 | 3 | 4 | 5 |
| --- | --- | --- | --- | --- | --- |
| 1.SN | **0.806** |  |  |  |  |
| 2.AD | 0.470** | **0.861** |  |  |  |
| 3.T | 0.563** | 0.417** | **0.826** |  |  |
| 4.CI | 0.354** | 0.215** | 0.615** | **0.709** |  |
| 5.E | 0.398** | 0.286** | 0.585** | 0.368** | **0.805** |

**Supplementary Table 3**. Correlations Between Variables.

Note. Squared root of AVE values are noted on the diagonal with bold. *

Correlation is significant at .05 level. **Correlation is significant at the .01 level (two-tailed).

| Index | x2 | x2/df | GFI | RMSEA | NFI | TLI | CFI |
| --- | --- | --- | --- | --- | --- | --- | --- |
| Result | 160.23 | 1.470 | 0.948 | 0.037 | 0.952 | 0.980 | 0.984 |

**Supplementary Table 4**. Fit Statistics for Model.

| Hypotheses | Construct | Path | Construct | Estimate | S.E. | C.R. | P | Result |
| --- | --- | --- | --- | --- | --- | --- | --- | --- |
| H1 | T | <--- | SN | 0.296 | 0.052 | 5.678 | *** | Support |
| H2 | T | <--- | CI | 0.591 | 0.084 | 7.067 | *** | Support |
| H3 | T | <--- | AD | 0.161 | 0.048 | 3.354 | *** | Support |
| H4 | T | <--- | SN*CI | 0.391 | 0.195 | 2 | 0.045 | Support |
| H5 | E | <--- | T | 0.491 | 0.051 | 9.698 | *** | Support |

**Supplementary Table 5**. Unstandardized Path Coefficients to Testing the Causal Effects of the Constructs for Model.

| Hypotheses | Path | Estimate | Boot  S.E． | Bias-corrected 95%CI | | P | Result |
| --- | --- | --- | --- | --- | --- | --- | --- |
|  |  |  |  | Lower | Upper |  |  |
| H6 | SN→T→E | 0.195 | 0.044 | 0.117 | 0.285 | 0.001 | Support |
| H7 | AD→T→E | 0.105 | 0.037 | 0.035 | 0.179 | 0.005 | Support |

**Supplementary Table 6.** Bootstrap test for significance of mediating effects.

|  | Effect | S.E. | t-value | p | LLCI | ULCI |
| --- | --- | --- | --- | --- | --- | --- |
| constant | 3.6004 | 0.0361 | 99.8659 | 0 | 3.5295 | 3.6713 |
| SN | 0.4023 | 0.0427 | 9.4237 | 0 | 0.3183 | 0.4862 |
| CI | 0.4749 | 0.053 | 8.9635 | 0 | 0.3707 | 0.5791 |
| SN*CI | 0.1639 | 0.0677 | 2.4213 | 0.016 | 0.0308 | 0.2971 |

**Supplementary Table7.** Moderating effects testing.
